# Tractography patterns of pedunculopontine nucleus deep brain stimulation

**DOI:** 10.1007/s00702-021-02327-x

**Published:** 2021-03-29

**Authors:** Ashley L. B. Raghu, Tariq Parker, Amir P. Divanbeighi Zand, Stephen Payne, Jesper Andersson, John Stein, Tipu Z. Aziz, Alexander L. Green

**Affiliations:** 1 Oxford Functional Neurosurgery, Nuffield Department of Surgical Sciences, University of Oxford, Oxford, UK; 2 Department of Engineering, Institute of Biomedical Engineering, University of Oxford, Oxford, UK; 3 Nuffield Department of Clinical Neurosciences, Wellcome Centre for Integrative Neuroimaging, FMRIB, University of Oxford, Oxford, UK; 4 Department of Physiology, Anatomy and Genetics, University of Oxford, Oxford, UK; 5 Department of Neurosurgery, John Radcliffe Hospital, Oxford University NHS Foundation Trust, Oxford, UK

**Keywords:** Parkinson’s disease, Gait, Falls, Deep brain stimulation, Pedunculopontine nucleus, Pedunculotegmental nucleus, Tractography

## Abstract

Deep brain stimulation of the pedunculopontine nucleus is a promising surgical procedure for the treatment of Parkinsonian gait and balance dysfunction. It has, however, produced mixed clinical results that are poorly understood. We used tractography with the aim to rationalise this heterogeneity. A cohort of eight patients with postural instability and gait disturbance (Parkinson’s disease subtype) underwent pre-operative structural and diffusion MRI, then progressed to deep brain stimulation targeting the pedunculopontine nucleus. Pre-operative and follow-up assessments were carried out using the Gait and Falls Questionnaire, and Freezing of Gait Questionnaire. Probabilistic diffusion tensor tractography was carried out between the stimulating electrodes and both cortical and cerebellar regions of a priori interest. Cortical surface reconstructions were carried out to measure cortical thickness in relevant areas. Structural connectivity between stimulating electrode and precentral gyrus (*r* = 0.81, *p* = 0.01), Brodmann areas 1 (*r* = 0.78, *p* = 0.02) and 2 (*r* = 0.76, *p* = 0.03) were correlated with clinical improvement. A negative correlation was also observed for the superior cerebellar peduncle (*r* = −0.76, *p* = 0.03). Lower cortical thickness of the left parietal lobe and bilateral premotor cortices were associated with greater pre-operative severity of symptoms. Both motor and sensory structural connectivity of the stimulated surgical target characterises the clinical benefit, or lack thereof, from surgery. In what is a challenging region of brainstem to effectively target, these results provide insights into how this can be better achieved. The mechanisms of action are likely to have both motor and sensory components, commensurate with the probable nature of the underlying dysfunction.

**Supplementary Information:**

The online version contains supplementary material available at 10.1007/s00702-021-02327-x.

## Introduction

Gait freezing and balance dysfunction leading to falls are symptoms of Parkinson’s disease (PD) that are typically unresponsive to dopamine agonists or deep brain stimulation (DBS) of the subthalamic nucleus. Severity of these symptoms correlate with cholinergic deficits (Bohnen et al. 2009; Gilman et al. 2010), signalling that degenerating cholinergic nuclei, such as the pedunculopontine nucleus (PPN), may have a role (Rinne et al. 2008). The possibility of targeting the PPN for PD treatment was propelled by the demonstration of akinesia improvement in the MPTP monkey model of PD. This was achieved through microinjection of the GABA antagonist bicuculine into the PPN (Nandi et al. 2002), and through low-frequency electrical stimulation (Jenkinson et al. 2004), both independent of dopaminergic mechanisms (Jenkinson et al. 2006). Subsequently, both neurophysiological and functional imaging experiments have confirmed the crucial role of the PPN in control of locomotion, with different regions of the PPN involved in different aspects (Caggiano et al. 2018; Karachi et al. 2012; Tattersall et al. 2014).

Although neurosurgery centres have reported mixed clinical results from DBS targeting the PPN for postural instability and gait disturbances (PIGD) in PD (Ferraye et al. 2010; Thevathasan et al. 2011a), it is nonetheless clear that stimulation of the PPN region (ventrolateral pontine tegmentum) can alter aspects of gait (Thevathasan et al. 2012) and balance (Perera et al. 2018), at least in some patients. It remains unknown why the PIGD symptoms of some patients improve with treatment, and with others it does not. There is a lack of consensus on where and how to target the PPN, with anatomical variability of the brain stem between different patients compounding the challenge. This problem likely contributes to observed variability in outcomes. Recently, the connectomics approach to DBS has become established, repeatedly demonstrating its power to explain and predict the effects of DBS (Vanegas-Arroyave et al. 2016). The reasoning for its employment is three-fold, (a) stimulation is likely to modulate the functioning of regions with which it is structurally connected, (b) regional anatomy can be ‘fingerprinted’ by its connectivity profile, giving an individualised (non-atlas based) approach to identifying targets not readily discernible from imaging contrast, and (c) connectivity estimates between known regions can give insights into individual pathology and evolution of disease. Given the current uncertainties concerning PPN DBS, connectomic data is therefore highly relevant. Current data are limited to PPN tractography in healthy individuals (Muthusamy et al. 2007) and single DBS cases (Schweder et al. 2010), leaving questions relating to clinical outcomes unaddressed.

We have therefore used structural and diffusion MRI in a series of PIGD-PD patients who underwent DBS targeting the PPN, to understand the relationship between tractography-based structural connectivity estimates of stimulated regions in individuals, and their clinical outcomes following surgery. We followed up implications of our results by analysing relevant regions of cortical thickness (as an indicator of atrophy) with respect to pre-operative symptom severity.

## Methods

### Patients

Between 2010 and 2012, eight consecutive patients (all male) with severe, medically refractory PIDG-PD were scheduled for implantation of bilateral electrodes (Medtronic 3887/89) in the PPN, undergoing surgery as described elsewhere (Thevathasan et al. 2011a), at the John Radcliffe Hospital. Briefly, we aim just lateral to the horizontal superior cerebellar decussating fibres in the pons. Respecting the lateral ventricles, as vertical as possible a trajectory is taken, with the purpose of passing the electrode through this point along the long axis of the PPN. All patients were referred to Oxford Functional Neurosurgery, met UK PD Brain Bank criteria, and underwent assessment by a consultant neurologist, neurosurgeon and neuropsychologist, all with expertise in movement disorders, before being offered surgical treatment.

### Questionnaires

The Gait and Falling Questionnaire (GFQ; score/64) was completed by patients pre-operatively and at 1–2 year follow-up (Giladi et al. 2000). The GFQ, and the Freezing of Gait Questionnaire (FOGQ; score/24), a component of the GFQ, were both analysed. Unified Parkinson’s Disease Rating Scale data were not analysed, as the corresponding PIGD metrics were deemed too insensitive (Thevathasan et al. 2011a; Welter et al. 2015).

### Diffusion imaging acquisition and pre-processing

Pre-operative MRI was performed on a 1.5 T Phillips Achieva using a modified spin-echo sequence with SENSE parallel imaging. In plane resolution was 1.818 × 1.818 mm^2^, and 64 2 mm thick slices were acquired in an interleaved fashion. Diffusion weighting (*b* = 1200 s/mm^2^) was applied along 32 non-colinear gradient directions, with one non-diffusion weighted volume (*b* = 0). Correction for distortions and subject movement was carried out using the *FMRIB Software Library* (FSL; Oxford, UK) (Smith et al. 2004). The susceptibility-induced off-resonance field was estimated using *topup* (Andersson et al. 2003; Smith et al. 2004). Instead of using two *b* = 0 spin-echo EPI with opposing PE-direction, the field was estimated from a *b* = 0 volume and a structural T2-weighted scan, without any distortions. Motion and eddy currents were corrected for using *eddy* (Andersson and Sotiropoulos 2016) with outlier detection and replacement (Andersson et al. 2016). Single shell ball and stick modelling of local diffusion parameters was carried out using BEDPOSTX, with up to two crossing fibres per voxel (Behrens et al. 2003).

### Deep brain stimulation

Patients were programmed as described elsewhere to maximise improvements in gait and imbalance (Thevathasan et al. 2011a). Briefly, stimulation from all contacts was systematically explored, beginning with monopolar screening at 35 Hz, 60 μs and amplitude titrated with ceilings established by side effects (e.g. oscillopsia). Bipolar stimulation was explored for additional benefit/tolerability.

### Tractography

Probabilistic tractography was carried out using PROBTRACX (Behrens et al. 2003).

### Termination masks

A T1-weighted pre-operative image was used to generate parcellated anatomic surfaces using *Freesurfer* (Harvard, USA) (Fischl 2012). T2 fluid-attenuated inversion recovery (FLAIR) images were used for pial surface optimization. Parcellations of the white–grey matter boundary surface were utilised as masks, including the precentral gyrus and postcentral gyrus from the Desikan Kelliany Atlas (Desikan et al. 2006), as well as BA1, 2, 3a, 3b and 6. As no supplementary motor area (SMA) parcellation was available, the Harvard–Oxford cortical atlas was used to generate a mask of the SMA, which was then registered to MRI scans using FLIRT (Smith et al. 2004). The ICBM-DTI-81 white matter labels atlas was used to generate a mask of the superior cerebellar peduncle (SCP), which was cropped to the cerebellar portion only, and registered to MRI scans using FLIRT and FNIRT (Andersson et al. 2007; Smith et al. 2004).

### Cathode and volume of activated tissue (VAT)

Post-operative computed tomography (CT) images were registered to MRI using FLIRT (Smith et al. 2004). Lead contacts were identified based on CT artefacts and array dimensions. VAT around the cathode was approximated as a sphere, calculated based on a finite element model, utilising impedance and voltage data from the DBS system acquired at follow-up (Mädler and Coenen 2012). VAT is based on axonal activation. However, the precise mechanisms of DBS in the PPN region are unknown. As such, the cathode contact for each lead was represented as a single diffusion voxel; single-voxel seed analysis has value as the centroid of any other effects, and for possible targeting implications. VAT masks were seeded and tracked to each termination mask. Stimulation cathode masks were seeded and tracked to precentral gyrus, BA6, SMA and SCP termination masks only. ‘ <–> ’ is used to denote streamlines between seed and termination mask.

### PPN region

To assess whether our cathode <–> SMA results were driven by the cathode’s precise location, tractography from the larger PPN region was carried out. An 8 mm column (four diffusion voxels), descending from the mid-inferior collicular level, was used to represent the PPN region. Placement was reviewed and agreed on by stereotactic surgeons TZA and ALG. PPN region masks were seeded and tracked to the SMA termination mask only.

### Statistics

For each of the streamlines generated by PROBTRACX, we counted the number of seeds which reached each of the regions described above. Those counts were explored for linear relationships with clinical GFQ and FOGQ improvement (absolute) by calculating both Pearson and Spearman’s rho correlations (two-tailed) in SPSS (IBM, New York). Stimulation cathode and VAT connectivity were averaged over left and right for each patient, then compared with the difference between pre-operative and follow-up questionnaire scores. Statistically significant relationships were assessed further by substituting relative (%) clinical improvement as the dependent variable.

### Electrode locations

The vertical distance between stimulating cathode and the obex was calculated from fused CT-FLAIR, and plotted against associated clinical outcomes. Leads were plotted in MNI ICBM 2009b NLIN ASYM space using LeadDBS v2.3. (Horn et al. 2019) Coregistration was performed with FLIRT, normalisation with ANT, electrode reconstruction with PaCER, and plotted with nuclei from the Harvard AAN atlas, in Lead Group.

### Cortical thickness

Respecting both somatosensory and motor aspects of the tractography results, cortical thickness of respective functional domains were analysed as an atrophy surrogate. Mean thickness values were extracted from Freesurfer’s statistical output for BA 1, 2, 3a, 3b and 6, precentral gyrus, postcentral gyrus, supramarginal gyrus, superior and inferior parietal regions and subjected to correlational analysis, both unilaterally and bilaterally, with the severity of pre-operative symptoms (GFQ).

### Standard protocol approvals, registrations, and patient consents

Research approval was obtained by a local ethics committee (NRES SOUTH CENTRAL OXFORD A 08/H0604/58). Patient’s consent was obtained according to the Declaration of Helsinki.

## Results

Seven of eight patients were successfully implanted with DBS electrodes bilaterally. In one patient, one electrode was not included due to its unsatisfactory position. Three of eight patients showed deterioration at follow-up (Table 1, median = 12 m, Q1–Q3: 12–16 m). Adverse outcomes from surgery included a small subdural haematoma in patient C, post-operative confusion (Salmonella) in patient E, and a post-operative diplopia on left lateral gaze in patient F, that resolved by discharge. Side effects of stimulation which bounded stimulation parameters (Table 2) included oscillopsia, buzzing in head/eye/nose, tightness around head, tightening of jaw, contralateral arm tremor, and pulling in the eye. Patient H developed a mild dysarthria associated with deep breathing, which may have been related to stimulation.

**Table 1:** Clinical data

Patient	Age at Surgery	Qu	Pre-op	Improvement		Follow up /mon	Long term outcome
				Absolute	%		
A	55	GFQ	55	14	25	12	Still uses at 10 years
		FOGQ	22	7	32		
B	77	GFQ	39	5	13	12	Dead 4 years after surgery
		FOGQ	22	5	23		
C	74	GFQ	22	1	5	12	Dead 3 years after surgery
		FOGQ	15	6	40		
D	56	GFQ	40	2	5	12	Dead 8 years after surgery
		FOGQ	15	2	13		
E	67	GFQ	30	−14	−47	18	Dead 4 years after surgery
		FOGQ	11	−9	−82		
F	71	GFQ	30	−8	−27	29^a^	Dead 4 years after surgery
		FOGQ	11	−3	−27		
G	68	GFQ	43	−3	−7	16	Revision at 18 months, no benefit. Doesn’t use
		FOGQ	21	0	0		
H	71	GFQ	36	12	33	12	Dead 6 years after surgery
		FOGQ	13	−1	−8		

*Qu *questionnaire, *Pre-op *pre-operative

^a^Follow-up delayed due to patient illness

**Table 2:** Stimulation parameters

Patient	Side	Frequency/Hz	Amplitude/V	Pulse width/μs
A	Left	35	2	60
	Right	35	2	60
B	Left	NA	NA	NA
	Right	35	2	60
C	Left	35	3.5	60
	Right	35	3.5	60
D	Left	35	3.5	70
	Right	35	2.8	90
E	Left	35	4	90
	Right	35	4	90
F	Left	25	4.3	70
	Right	25	4.3	70
G	Left	35	2.5	90
	Right	35	2.5	90
H	Left	35	3.8	60
	Right	35	4	60

### Tractography

**Fig. 1:**
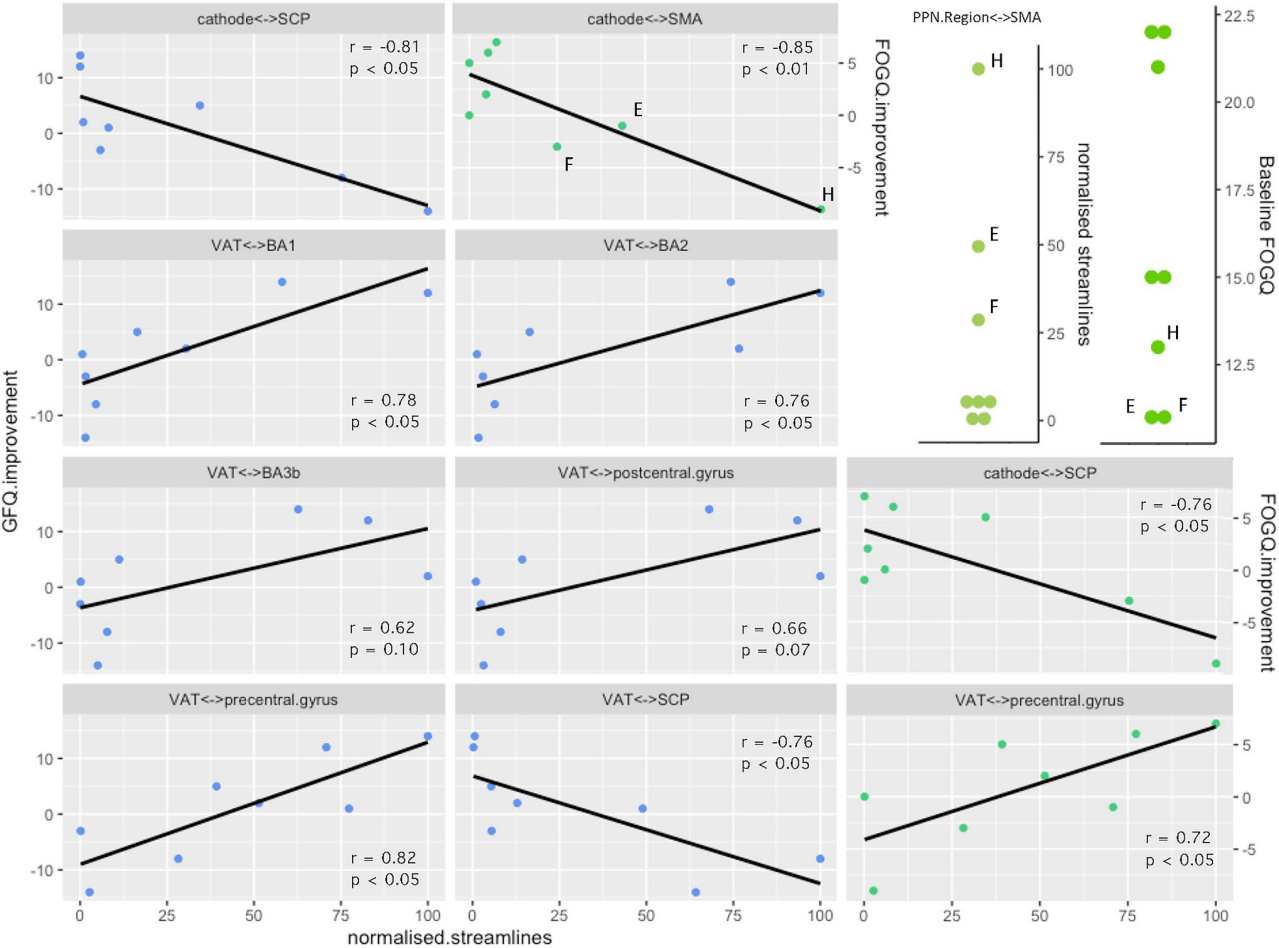
Tractography. Correlation of clinical improvement with structural connectivity between DBS leads and cortical/cerebellar regions of interest. Pearson: see panels. GFQ Spearman’s: Cathode <–> SCP: *r* = −0.81, *p* < 0.05. VAT <–> BA1: *r* = 0.79, *p* < 0.05. VAT <–> BA2: *r* = 0.71, *p* < 0.05. VAT <–> BA3b: r = 0.64, *p* = 0.09. VAT <–> postcentral gyrus: *r* = 0.62, *p* = 0.10. VAT <–> precentral gyrus: *r* = 0.76, *p* < 0.05. VAT <–> SCP: *r* = −0.88, *p* < 0.01. FOGQ Spearman’s: Cathode <–> SMA: *r* = −0.55, n.s. Cathode <–> SCP: *r* = −0.45, n.s. VAT <–> precentral gyrus: *r* = 0.71, *p* < 0.05. Top right: Patients E, F and H PPN Region <–> SMA connectivity and baseline FOGQ. Normalised streamlines = streamline count normalised to maximum in series as 100

Under parametric analysis, VAT connectivity with four regions (precentral gyrus, SCP, BA1, BA2) demonstrated significant (*p* < 0.05) relationships with clinical GFQ outcomes (Fig. 1). VAT <–> precentral gyrus connectivity, alone, demonstrated significant relationships with both GFQ and FOGQ improvement. All these survived non-parametric assessment (Fig. 1). Cathode connectivity with two regions demonstrated significant parametric relationships with clinical outcomes (SCP: GFQ and FOGQ, SMA: FOGQ only), but neither FOGQ correlation reached significance under non-parametric assessment (Fig. 1). The negative correlation of cathode/VAT <–> SCP connectivity with GFQ improvement was not mediated by a correlation with either SCP fractional anisotropy (FA) (r = 0.01, n.s) or mean diffusivity (*r* = 0.28, n.s). Connectivity with BA6 did not demonstrate any significant relationships with clinical improvement (GFQ. VAT: *r* = 0.47, n.s; cathode: *r* = 0.53, n.s), likewise between VAT and postcentral gyrus, BA3b (Fig. 1), or 3a (*r* = 0.22, n.s).

Most significant findings for absolute change in clinical outcome remained significant when assessed against relative (%) change (VAT-GFQ: precentral *r* = 0.77, SCP *r* = −0.78, BA1 *r* = 0.75, BA2 *r* = 0.72; Cathode-GFQ: SCP *r* = −0.87; Cathode-FOGQ: SCP *r* = −0.83, SMA *r* = −0.90). Correlation of relative FOGQ improvement and VAT <–> precentral gyrus connectivity became insignificant (*r* = 0.69, *p* = 0.056).

### Cortical thickness

**Fig. 2:**
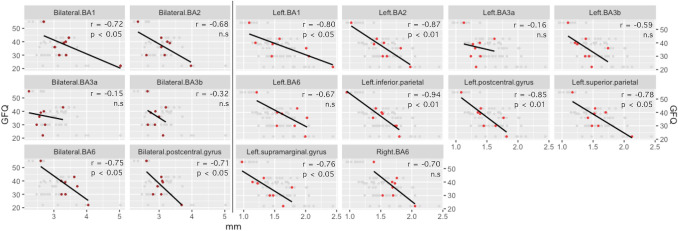
Cortical thickness. Correlation of mean cortical thickness with pre-operative symptom severity (GFQ). Pearson: see panels. Spearman’s: Bilateral*: BA1: r = −0.40, n.s. BA2: r = -0.49, n.s. BA3a: r* = −0.06, n.s. BA3b: *r* = −0.17, n.s. BA6: *r* = −0.58, n.s. postcentral gyrus: *r* = −0.31, n.s. Left: BA1: *r* = −0.72, *p* < 0.05. BA2: *r* = −0.77, *p* < 0.05. BA3a: *r* = −0.08, n.s. BA3b: *r* = −0.67, n.s. BA6: *r* = −0.58, n.s. inferior parietal: *r* = −0.85, *p* < 0.01. postcentral gyrus: *r* = −0.85, *p* < 0.01. superior parietal: *r* = −0.44, n.s.. Left supramarginal: *r* = −0.78, *p* < 0.05. Right: BA6: *r* = −0.37, n.s.

Under parametric analysis, assessed bilaterally, BA6, BA1 and the postcentral gyrus demonstrated significant (*p* < 0.05) negative correlations with pre-operative GFQ (Fig. 2). BA2 trended to significance (*p* = 0.06). Assessed unilaterally, on the right, only BA6 trended to significance in this regard (*p* = 0.053). On the left, BA1, BA2, postcentral and supramarginal gyri, inferior and superior parietal regions demonstrated significant (*p* < 0.05) negative correlations with pre-operative GFQ (Fig. 2). BA2 (*p* = 0.06) and BA6 (*p* = 0.07) trended to significance. No regions, assessed bilaterally, survived non-parametric testing for significance. Left BA1, BA2, left postcentral, supramarginal, inferior parietal remained significant under non-parametric assessment, and BA3b trended to significance (*p* = 0.07).

### Electrode locations

**Fig. 3:**
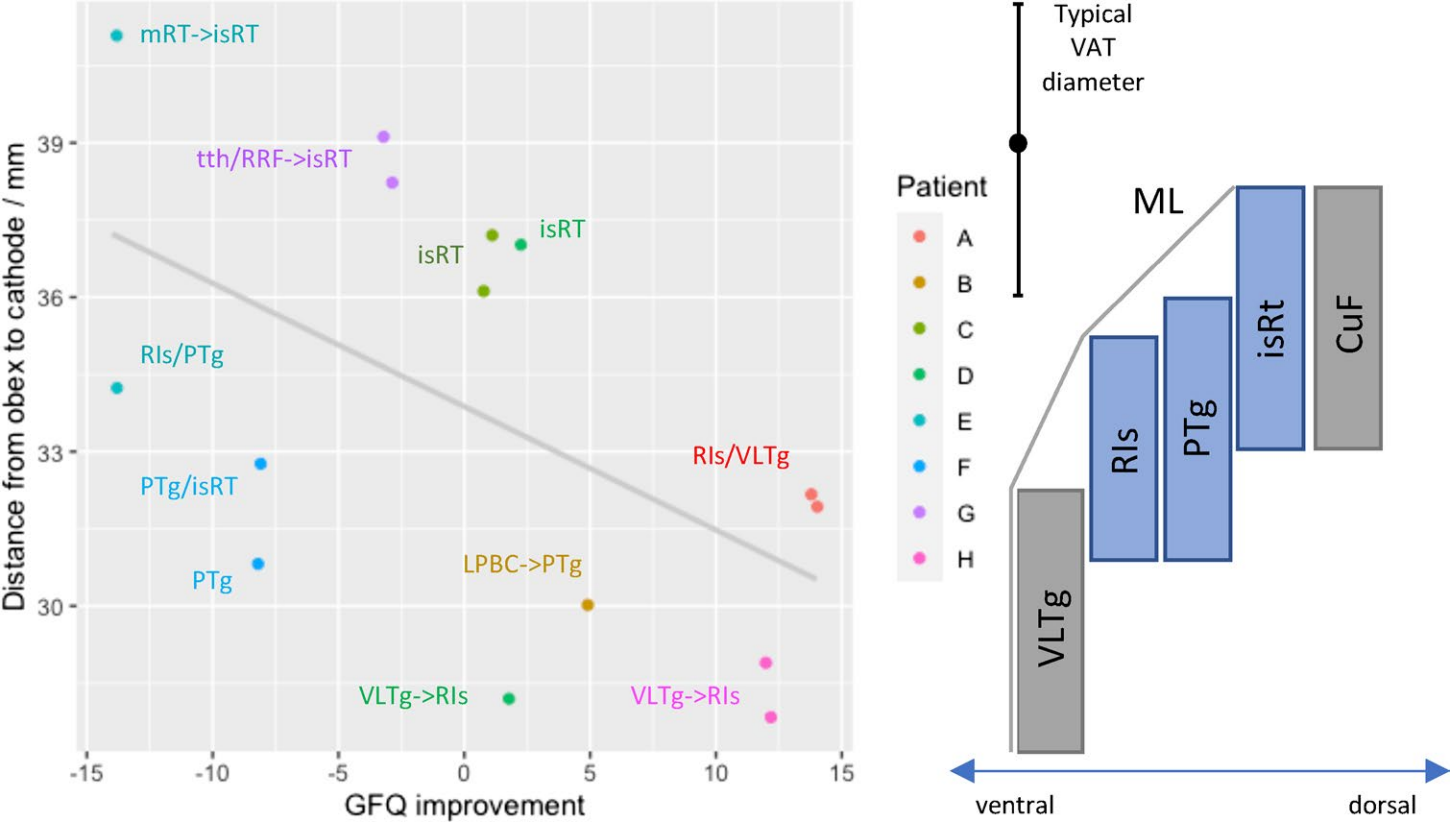
Cathode depth. Plot of depth versus clinical improvement (left), contextualised relative to nuclei of the ventrolateral pontine tegmentum by Paxinos et al. (2012) brainstem atlas (right: vertical axis to plot’s scale, ventral-dorsal axis not to scale), and grounded in distance to the obex. Cathodes are labelled with the most likely location within the PPN based on the same atlas. When distance from the obex precludes atlas-based assignment to the PPN, ‘- > ’ denotes most likely PPN location to be stimulated. VLTg = ventrolateral tegmental area; Ris = retroisthmic nucleus; PTg = pedunculotegmental nucleus; isRt = isthmic reticular formation; CuF = cuneiform nucleus; ML = medial lemniscus; mRT = mesencephalic reticular formation; tth = trigeminal lemniscus; RRF = retrorubral field; LPBC = lateral parabrachial complex; VAT = volume of activated tissue

**Fig. 4:**
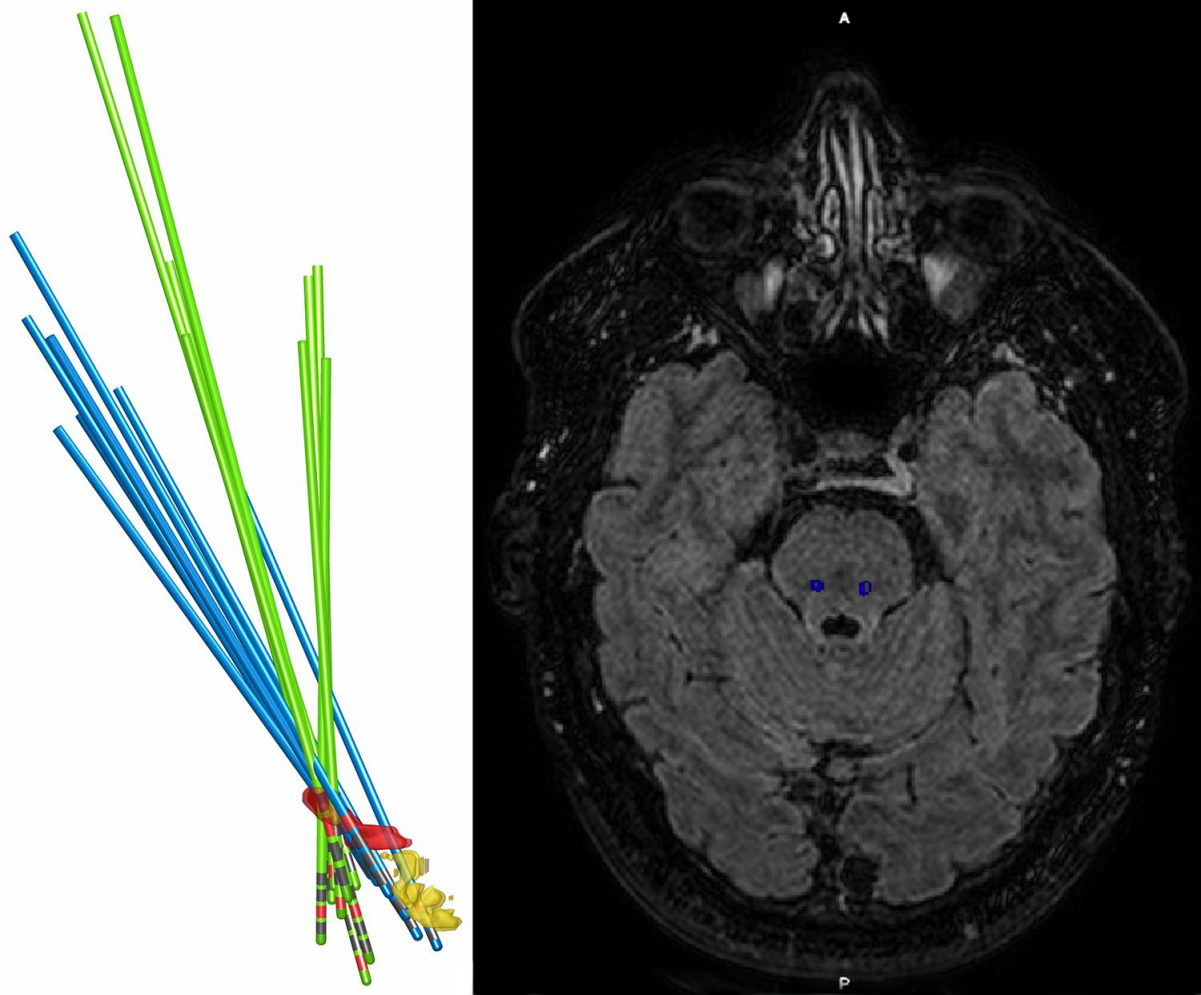
Stimulation electrodes. Left: Reconstruction of DBS electrodes. Blue = Improved gait freezing. Green = No improvement. Red = PPN. Yellow = Parabrachial nuclei. Right: Fused CT-FLAIR showing stimulating cathode locations of Patient A

A trend was observed for deeper stimulation to be more effective (Fig. 3). DBS lead trajectories were less oblique for patients whose FOGQ scores had not improved at follow-up (Fig. 4).

## Discussion

### Anatomy

The ventrolateral pontine tegmentum is a cytoarchitecturally and functionally complex region, leading to varying accounts of its parcellation and nomenclature. The PPN (Lavoie and Parent 1994) or pedunculopontine tegmental nucleus (PPTg) (Paxinos and Huang 1995) divided into compacta and dissipata subnuclei, has more recently been revised to three nuclei: the pedunculotegmental nucleus (PTg), isthmic reticular formation (iTR) and the retroisthmic nucleus (RIs) (Paxinos et al. 2012). At the caudal pole of this complex, the thin ventrolateral tegmental area (VLTg) separates the RIs from the medial lemniscus (ML), and continues caudally as a larger structure than previously described (Paxinos and Huang 1995; Paxinos et al. 2012). Mazzone et al. have identified the RIs/VLTg (ventral-caudal PPN) as a likely location of beneficial stimulation in their patients (Mazzone et al. 2016).

### Electrode locations

Our results are somewhat consistent with the above. A trend was observed for deeper stimulation being better (Fig. 3). In addition, the somatosensory and cerebellar tractography results (Fig. 1) are suggestive of a ventrolateral tendency of effective stimulation, as the PPN is bounded in that respect by the ML and spinothalamic tract, which both project strongly to the postcentral gyrus. That the caudal and rostral PPN should be distinguished is now clear. There are major chemical and structural differences, for example, GABAergic neurons are much more populous in the caudal PPN (Martinez-Gonzalez et al. 2011) and structural connectivity with motor cortices is weaker there (Matsumura et al. 2000). There is also clinical (Ferraye et al. 2010; Thevathasan et al. 2011a) and pre-clinical support for the caudal PPN as the preferable target for ameliorating gait disorders (Gut and Winn 2015).

Patients with improvement in gait freezing, had lead implants following more anterior–posterior oblique trajectories, consistent with the PPN long-axis (Fig. 4). DBS lead implantation alone can be considered a microlesion intervention along its trajectory, with accompanying peri-lead gliosis. Step length and speed have been observed to improve after surgery, in the absence of stimulation, and so has been attributed to a lesioning effect (Welter et al. 2015). As such, disruption of the PPN by implantation through its length, could be a critical feature of successful surgery to improve gait freezing. As a practical consideration, however, when large lateral ventricles are present, as is often the case in these patients due to atrophy, trajectories to access the pontine tegmentum can be very limited.

### Tractography

We had good a priori reasons to justify investigating structural connectivity with the regions of interest in our study. Neuronal tracer studies have identified frontal cortical projections to the PPN, in particular from primary motor, and premotor cortices (Matsumura et al. 2000; Monakow et al. 1979). Considering the motor phenomenology of falls and gait disturbance, examining the precentral gyrus and BA6 is clearly justified. Cerebellar connectivity was also assessed, namely the SCP, as both afferent and efferent connections have been established by neuronal tracing (Hazrati and Parent 1992) and the well-established importance of the cerebellum in motor control and balance. The PPN is located between three ascending sensory pathways: the spinothalamic tracts, the SCP, and perhaps most importantly, the ML. Considering both the proximity of the PPN to the ML, and the typical VAT (radius ≈ 3–4 mm), this implies that when an electrode is placed in the PPN, capture of the ascending lemniscal system in the tractography seed is highly likely (see Paxinos et al. 2012 for anatomical distances). Postcentral gyrus connectivity merited investigation, a) as a marker of accurate placement in the PPN, and b) the possibility that incidental ML stimulation may contribute to clinical improvement. Some of our patients reported somatosensory paraesthesias with increasing amplitude of low-frequency stimulation, and there is credible neurophysiological evidence that chronic PPN DBS does reversibly neuromodulate the ML (Mazzone et al. 2016) Furthermore, evidence is accumulating that suggests dorsal column stimulation improves Parkinsonian gait dysfunction and falls (Samotus et al. 2018). Therefore, although direct anatomical connections between the parietal lobe and PPN have not been established, and neurophysiological evidence for such connections is weak (Insola et al. 2011) we examined connectivity between the VAT (but not the cathode) and the postcentral gyrus, as well as its functionally distinct divisions (BA 3a, 3b, 1, 2).

Connectivity of the crown (BA1) and posterior bank (BA2) of the postcentral gyrus had a stronger correlation with improvement than the anterior bank (BA3a/b). If one posits a causal relationship between ML modulation and clinical improvement, one might expect that BA2 connectivity would be highly correlated. BA2 projects to primary motor cortex (Porter 1992) but also receives proprioceptive input, which it combines with tactile information (Keysers et al. 2010). Left and right BA2 also have dense reciprocal callosal projections (Killackey et al. 1983), indicating a function in sided coordination. The alternative interpretation is that the positive correlation *only* represents a marker of the PPN lead being located correctly, or otherwise. Explicitly, this suggests optimal implantation in the ventral PPN (or the VLTg), i.e. adjacent to the ML, a region that other experienced authors, with some of the best clinical results, have commonly located their electrodes (Mazzone et al. 2016).

The correlation of VAT <–> precentral gyrus connectivity with clinical improvement was robust. High connectivity is likely to be a feature of successful PPN-DBS, which emphasises the importance of precentral gyrus input to the PPN. Stimulation of the PPN region can elicit locomotion in decerebrate animals, *ipso facto*, without cortical input (Garcia-Rill et al. 1987), and in PIGD-PD there is a block to the release of pre-programmed ballistic movements, which can be relieved by PPN stimulation (Thevathasan et al. 2011b). It is possible cortical modulation of the diseased PPN may function as a block to subcortically and spinally located locomotor programmes, which low-frequency stimulation can release. Chronic dopamine depletion in a rodent model of PD demonstrated the development of strengthened, abnormal low-frequency functional connectivity between primary motor cortex and the PPN, led by the cortex (Valencia et al. 2014). This in principle demonstrates a pathological neurophysiological substrate which PPN DBS could ameliorate, and that is consistent with our tractography results. Conversely, in PD, low-frequency functional connectivity between the PPN region and the SMA arises during movement preparation on dopaminergic medications, but not off them (Tsang et al. 2010). Additionally, during motor performance, blood flow to the SMA is increased when PD akinesia is treated with dopamine agonists (Jenkins et al. 1992). This describes an opposing valence of precentral gyrus- and SMA-PPN interaction, that mirrors the motor cortex tractography correlations we found for gait freezing.

The SMA correlation with gait freezing we observed was driven by three patients (E, F, H) who deteriorated following surgery, and had much higher cathode <–> SMA connectivity (Fig. 1). While impossible to exclude, it is difficult to ascribe their high SMA connectivity to placement in regions outwith the PPN with potentially higher SMA connectivity, for example the cuneiform nucleus or retrorubral field. Supporting this, all three patients had high PPN region <–> SMA connectivity (Fig. 1), i.e. not just high SMA connectivity with the smaller locus where the cathode ended up. Furthermore, these patients also had the lowest pre-operative gait freezing severity (Fig. 1; Table 1), lending support to the notion that they have a different phenotype. Recognising that PD gait freezing can likely arise from different pathophysiological processes, it is plausible that when high connectivity between SMA and the PPN is present, that this is protective against gait freezing, and DBS is liable to disrupt it or otherwise leave the principal cause of freezing unchecked. Physiological top-down modulation of PPN output will depend on parallel, differential input from the SMA (e.g. posture preparation) and precentral gyrus (e.g. step initiation). It is possible that when this becomes uncoordinated or unbalanced, that the PPNs role in gait initiation is best served purely by subcortical circuits. This is perhaps consistent with findings that PPN DBS does not eliminate gait freezing, but can improve it in some patients.

While VAT modelling is derived from principles of axonal activation, results from cathode analysis may best represent other mechanisms, such as lesioning, whilst also offering a more precise connectivity mapping of the stereotactic target. Regarding the SCP results, high connectivity could derive from electrodes placed too medially, in the SCP. Irrespective of any true PPN <–> SCP connectivity variance, a negative correlation would be observed if electrodes were so positioned. If this is not responsible, it may be that beneficial cerebellar outflow (likely excitatory) to the PPN is disrupted by stimulation. However, as the SCP did not show signs of degeneration that explained differences in connectivity estimates, the former explanation seems most likely.

Overall, our results are consistent with the concept that gait dysfunction in PD has sensory and motor components: perhaps even implying a disorder of sensorimotor integration. Indeed, some authors have concluded that the PPN is best considered as a nucleus of sensorimotor integration (Winn 2008).

### Targeting and tractography

Targeting the PPN remains challenging and controversial (Mazzone et al. 2011). It is not clearly visible on typical MRI scans, and combined with its distance from the bicommissural plane and large non-linear inter-individual variation in brain stem anatomy, a consensus on targeting strategy remains to be reached (Hamani et al. 2016). Principle diffusion directions (Aravamuthan et al. 2007) and FA contrast have been proposed as useful for locating the PPN (Henssen et al. 2019), and we are grateful for the development and anatomic validation of an FA-based probabilistic atlas of the mesopontine tegmentum (Bianciardi et al. 2018). Nonetheless, recognising both the difficulty and uncertainties in targeting the PPN region, and the limitations in applying atlases, diffusion tractography offers an objective, ‘clinical outcome grounded’ approach to locating the optimum target in this region of the brainstem, in an individual patient. This may have the potential to supplant or operate as a modifier to targeting based on atlas-based anatomical relations. An alternative tractography-based approach would be to reconstruct the SCP, spinothalamic tract and ML (Cong et al. 2018) to help triangulate the PPN and demarcate a viable surgical corridor: a process which could be feasibly automated.

### Cortical thickness

Since both motor and somatosensory cortices appeared relevant to clinical improvement with stimulation, appealing to treatment-disease homology, it seemed plausible that atrophy of caudal-frontal and parietal lobes may relate to pre-operative symptom severity, measured by GFQ. Although we lacked longitudinal data, as cortical thickness in healthy brains has low variance in a given area, we supposed that cross-sectional analysis may nonetheless have some value. Our results suggest that left parietal lobe, bilateral postcentral gyrus, and bilateral premotor cortex atrophy may partly account for the PIGD-PD phenotype severity. This adds further support to the concept of gait dysfunction as both a motor and sensory failure. Remarkably, when considering divisions of the postcentral gyrus, the same pattern of correlation strength (BA3a < BA3b < BA1/2) was observed (Fig. 2), as it was with tractography (Fig. 1). This reinforces suspicion around the role of the posterior part of this gyrus in the dynamics of this disease. Cortical atrophy in PD has been previously studied, using both voxel-based morphometry and cortical thickness analyses (Herman et al. 2014; Uribe et al. 2018). Although specific results are mixed, both parietal lobe atrophy, and regions of BA6 (mostly medial) have been implicated in PIGD-PD. Loci of hypometabolism in the left postcentral gyrus and left inferior parietal lobule, detected by PET, have been observed to characterise PIGD-PD, distinguishing it from tremor-dominant PD (Zhang et al. 2016).

### Limitations

The key limitations of the study are primarily those intrinsic to tractography, our acquisition, and those related to cohort size. The latter puts large constraints on the power to detect relationships, and high likelihood of type II error. For example, we did not find that premotor cortex connectivity was important, although we suspected that it would be, given its well-established involvement in posture and gait. Nonetheless, that statistically significant relationships were found following investigation of a small number of a priori relevant structures, could be a testament to the importance of our findings.

Diffusion MRI tractography benefits from high numbers of gradient directions and the high angular resolution available from modern multi-shell acquisitions. Our diffusion data were acquired a decade ago, with a lower quality of structural information encoded in such data. Nonetheless, it is accepted that such a diffusion study can be used to produce meaningful tractography with meaningful clinical results (Vanegas-Arroyave et al. 2016). The same can be said of the utilised VAT approximation, even though it fails to account for theoretically important features, such as local fibre orientation.

Our field of view only included the brain, and did not extend down into the cervical spinal cord. In considering projections from the PPN, rodent data indicate that rostral connectivity is much larger than caudal connectivity (Spann and Grofova 1989). Nonetheless, diffusion data including the cervical spinal cord that could make a meaningful assessment of PPN-spinal cord connectivity would be attractive, as these circuits are thought to be important for the PPN's locomotor functions (Caggiano et al. 2018; Garcia-Rill et al. 1987; Skinner et al. 1990).

## Conclusions

DBS in the ventrolateral pontine tegmentum, targeting the PPN, is characterised by higher structural connectivity to the precentral gyrus and BA1/2 of the postcentral gyrus when it is effective in improving gait and balance symptoms of PIGD-PD. The converse can be said of connectivity to the SCP. Stimulating the most caudal and lateral part of the PPN may offer the best chance of relieving symptoms, whereas, stimulating the SCP medially may worsen them. Low cortical thickness of the left parietal lobe and bilateral premotor cortices is associated with higher severity of PIGD-PD disease. Together, this points to a sensorimotor mechanism of action and underlying dysfunction.

## Supplementary Information

Below is the link to the electronic supplementary material.

Supplementary file1 (DOCX 145 kb)

## Abbreviations

DBS: Deep brain stimulation
PPN: Pedunculopontine nucleus
PIGD: Postural instability and gait disturbance
GFQ: Gait and falling questionnaire
FOGQ: Freezing of gait questionnaire
