# Supplementary material for: Tractography patterns of pedunculopontine nucleus deep brain stimulation
Source: J Neural Transm (Vienna). 2021 Mar 29;128(5):659–70. doi: 10.1007/s00702-021-02327-x (PMC8105200; doi:10.1007/s00702-021-02327-x)
Supplement: Supplementary file 1 — Supplementary file1 (DOCX 145 kb) [file 702_2021_2327_MOESM1_ESM.docx]

Precentral

BA2

BA3a

BA1

BA3b


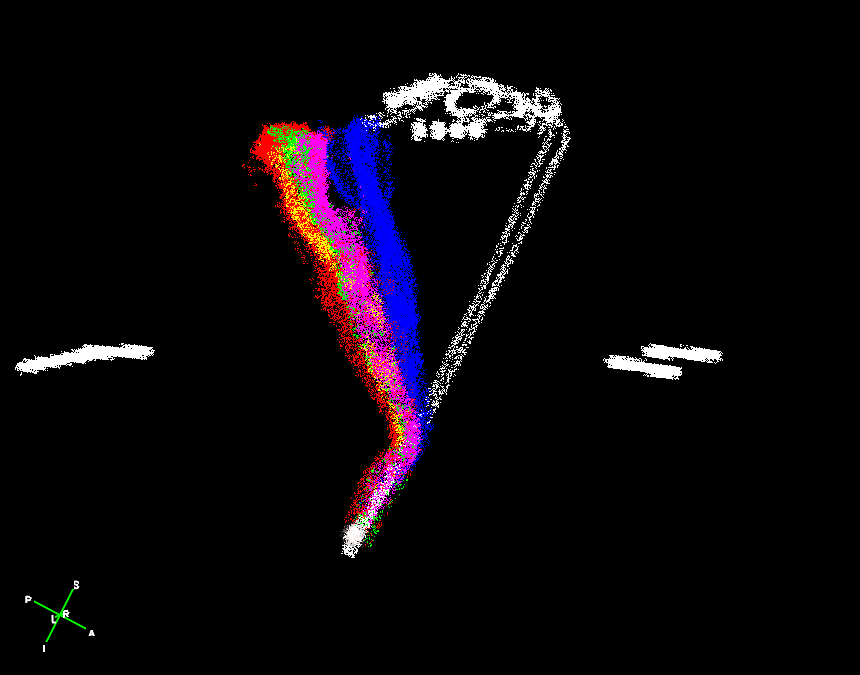


A

P

**Supplementary Figure 1.** Rendering of DBS leads and cortical probabilistic tractography
